# Supplementary material for: A druggable secretory protein maturase of Toxoplasma essential for invasion and egress
Source: eLife. 2017 Sep 12;6:e27480. doi: 10.7554/eLife.27480 (PMC5595437; doi:10.7554/eLife.27480)
Supplement: Supplementary file 1. — Predicted or experimentally validated subcellular localization is indicated. Gene IDs refer to ToxoDB, Release 31. [file elife-27480-supp1.docx]

**Supplementary File 1**. Proteins represented by peptides with normalized ATc+/ATc- abundance ratios <0.22 (stringent). Gene IDs refer to ToxoDB, Release 31.

| ToxoDB gene ID | Product | HMM SP probability | # TMs | Trafficking | TAILS features & interpretation |
| --- | --- | --- | --- | --- | --- |
| TGGT1_204050 | subtilisin SUB1 | 1 | 0 | secretory pathway | C-terminal processing after F145? (133-145<0.22 while the regular tryptic 133-157 >2). Processed N-term (no acetyl.) likely @ (G43) Y45 (0.036); several degrad prod. >2; SP: 1-24 |
| TGGT1_262730 | rhoptry protein ROP16 | 1 | 0 | secretory pathway | Strong evidence for acetylated N-term  @E29; SP: 1-24 |
| TGGT1_312270 | rhoptry protein ROP13 | 0.99 | 1 | secretory pathway | Strong evidence for processed N-term (acetyl.)  @65/67/68; SP: 1-19/27 |
| TGGT1_218520 | microneme protein MIC6 | 0.95 | 1 | secretory pathway | Strong N-term (no acetyl.), ratio 0.089  @ E95, SP: 1-24, |
| TGGT1_208030 | microneme protein MIC4 | 0.98 | 0 | secretory pathway | Peptides representing two N-termini (no acetyl.)  at I45 or S58 with similar support. SP: 1-27 |
| TGGT1_319560 | microneme protein MIC3 | 1 | 0 | secretory pathway | Strong evidence for processed N-term (acetyl.)  @A57; SP: 1-27 |
| TGGT1_204530 | microneme protein MIC11 | 0.99 | 0 | secretory pathway | Strong evidence for processed N-term (no acetyl.)  @E58; multiple degrad products >2; SP1: 1-23 |
| TGGT1_221180 | hypothetical protein | 0 .99 | 5 | secretory pathway | N-term at A 471 (no acetyl.) Acetyl. N-term peptide at A552 has no quant data. SP: 1-32 |
| **TGGT1_258360** | **hypothetical protein (TAILS5)** | **0.99** | **0** | **secretory pathway** | **Strong evidence for processed N-term (acetyl.)**  **@S83; SP: 1-20/29** |
| TGGT1_261740 | hypothetical protein | 0.93 | 1 | secretory pathway | N-term (acetyl:) @A52 (0.205); SP: 1-23 |
| TGGT1_204130 | perforin-like protein PLP1 | null | 0 | cytoplasmic | null |
| TGGT1_212210 | hypothetical protein | null | 0 | cytoplasmic | null |
| **TGGT1_273860** | **hypothetical protein (TAILS6)** | **null** | **0** | **cytoplasmic** | **null** |
| **TGGT1_279420** | **hypothetical protein (TAILS7)** | **null** | **0** | **cytoplasmic** | **null** |
| TGGT1_292920 | putative heat shock protein 75 | null | 0 | cytoplasmic | null |
| TGGT1_315270 | hypothetical protein | null | 0 | cytoplasmic | null |
| **TGGT1_321650** | **hypothetical protein (TAILS8)** | **null** | **1** | **cytoplasmic** | **null** |
