# Supplementary material for: A druggable secretory protein maturase of Toxoplasma essential for invasion and egress
Source: eLife. 2017 Sep 12;6:e27480. doi: 10.7554/eLife.27480 (PMC5595437; doi:10.7554/eLife.27480)
Supplement: Supplementary file 2. — Predicted or experimentally validated subcellular localization is indicated. Gene IDs refer to ToxoDB, Release 31. [file elife-27480-supp2.docx]

**Supplementary File 2**. Proteins represented by peptides with normalized ATc+/ATc- abundance ratios of <0.5 (relaxed). Predicted or experimentally validated subcellular localization is indicated. Gene IDs refer to ToxoDB, Release 31.

| ToxoDB gene ID | Product | HMM SP probability | # TMs | Trafficking |
| --- | --- | --- | --- | --- |
| TGGT1_308090 | rhoptry protein ROP5 | 1 | 0 | secretory pathway |
| TGGT1_243730 | rhoptry protein ROP9 | 0.6 | 1 | secretory pathway |
| TGGT1_312270 | rhoptry protein ROP13 | 0.99 | 1 | secretory pathway |
| TGGT1_262730 | rhoptry protein ROP16 | 1 | 0 | secretory pathway |
| TGGT1_311470 | rhoptry neck protein RON5 | 0.99 | 0 | secretory pathway |
| TGGT1_229010 | rhoptry neck protein RON4 | 1 | 0 | secretory pathway |
| TGGT1_291960 | rhoptry kinase family protein ROP40 (incomplete catalytic triad) | 0.83 | 0 | secretory pathway |
| TGGT1_319560 | microneme protein MIC3 | 1 | 0 | secretory pathway |
| TGGT1_208030 | microneme protein MIC4 | 0.98 | 0 | secretory pathway |
| TGGT1_277080 | microneme protein MIC5 | 0.98 | 1 | secretory pathway |
| TGGT1_218520 | microneme protein MIC6 | 0.95 | 1 | secretory pathway |
| TGGT1_204530 | microneme protein MIC11 | 0.99 | 0 | secretory pathway |
| TGGT1_260190 | microneme protein MIC13 | 1 | 0 | secretory pathway |
| TGGT1_289630 | microneme protein MIC16 | 0.94 | 1 | secretory pathway |
| TGGT1_270240 | MAG1 protein | 0.73 | 1 | secretory pathway |
| TGGT1_247520 | hypothetical protein | 0.81 | 1 | secretory pathway |
| TGGT1_221180 | hypothetical protein | 0.99 | 5 | secretory pathway |
| **TGGT1_258360** | **hypothetical protein (TAILS5)** | **0.99** | **0** | **secretory pathway** |
| TGGT1_261740 | hypothetical protein | 0.93 | 1 | secretory pathway |
| TGGT1_279100 | hypothetical protein | 0.95 | 1 | secretory pathway |
| TGGT1_310780 | dense granule protein GRA4 | 1 | 0 | secretory pathway |
| TGGT1_227620 | dense granule protein GRA2 | 1 | 0 | secretory pathway |
| TGGT1_290160 | putative sortilin | 0.59 | 1 | secretory pathway |
| TGGT1_215280 | succinate dehydrogenase [ubiquinone] iron-sulfur protein | 0.88 | 0 | secretory pathway |
| TGGT1_204050 | subtilisin SUB1 | 1 | 0 | secretory pathway |
| **TGGT1_202870** | **SAP domain-containing protein (TAILS1)** | **0.98** | **1** | **secretory pathway** |
| TGGT1_271050 | SAG-related sequence SRS34A | 1 | 0 | secretory pathway |
| TGGT1_410360 | putative transmembrane protein | 0.97 | 1 | secretory pathway |
| TGGT1_244560 | putative heat shock protein 90 | 1 | 1 | secretory pathway |
| TGGT1_212210 | hypothetical protein | null | 0 | cytoplasmic |
| TGGT1_213030 | hypothetical protein | null | 0 | cytoplasmic |
| TGGT1_215220 | hypothetical protein | null | 0 | cytoplasmic |
| TGGT1_249780 | hypothetical protein | null | 0 | cytoplasmic |
| TGGT1_253820 | hypothetical protein | null | 0 | cytoplasmic |
| **TGGT1_273860** | **hypothetical protein (TAILS6)** | **null** | **0** | **cytoplasmic** |
| **TGGT1_279420** | **hypothetical protein (TAILS7)** | **null** | **0** | **cytoplasmic** |
| TGGT1_293740 | hypothetical protein | null | 0 | cytoplasmic |
| TGGT1_315270 | hypothetical protein | null | 0 | cytoplasmic |
| **TGGT1_321650** | **hypothetical protein (TAILS8)** | **null** | **1** | **cytoplasmic** |
| TGGT1_309560 | nmda receptor glutamate-binding chain | null | 7 | cytoplasmic |
| TGGT1_286160B | non-specific serine/threonine protein kinase | null | 0 | cytoplasmic |
| TGGT1_277270 | NTPase II | null | 0 | cytoplasmic |
| TGGT1_243960 | nuclear transport factor 2 (ntf2) domain-containing protein | null | 0 | cytoplasmic |
| TGGT1_244110 | nucleosome assembly protein (nap) protein | null | 0 | cytoplasmic |
| TGGT1_204130 | perforin-like protein PLP1 | null | 0 | cytoplasmic |
| TGGT1_263060 | Proteasome/cyclosome repeat-containing protein | null | 0 | cytoplasmic |
| TGGT1_235020 | putative COPI protein | null | 0 | cytoplasmic |
| TGGT1_201680 | putative eukaryotic initiation factor-3 subunit 10 | null | 0 | cytoplasmic |
| TGGT1_292920 | putative heat shock protein 75 | null | 0 | cytoplasmic |
| TGGT1_236540 | RNA recognition motif-containing protein | null | 0 | cytoplasmic |
| TGGT1_264610 | RNA recognition motif-containing protein | null | 0 | cytoplasmic |
| TGGT1_265530 | RNA recognition motif-containing protein | null | 0 | cytoplasmic |
| TGGT1_321500 | RNA recognition motif-containing protein | null | 0 | cytoplasmic |
| TGGT1_318620 | RNA-directed RNA polymerase | null | 0 | cytoplasmic |
| TGGT1_319570 | WD domain, G-beta repeat-containing protein | null | 0 | cytoplasmic |
