# Supplementary material for: A druggable secretory protein maturase of Toxoplasma essential for invasion and egress
Source: eLife. 2017 Sep 12;6:e27480. doi: 10.7554/eLife.27480 (PMC5595437; doi:10.7554/eLife.27480)
Supplement: Supplementary file 3. — Predicted or experimentally validated subcellular localization is indicated. Gene IDs refer to ToxoDB, Release 31. [file elife-27480-supp3.docx]

**Supplementary File 3.** Proteins represented by peptides with normalized ATc+/ATc- abundance ratios of >2. Predicted or experimentally validated subcellular localization is indicated. Gene IDs refer to ToxoDB, Release 31.

| ToxoDB  gene ID | Product | HMM signal peptide probability | # TM Domains | Trafficking |
| --- | --- | --- | --- | --- |
| TGGT1_291960 | rhoptry kinase family protein ROP40 (incomplete catalytic triad) | 0.83 | 0 | secretory pathway |
| TGGT1_236210 | putative peptidase M16 family potein | 0.94 | 0 | secretory pathway |
| TGGT1_235620 | deoxyhypusine synthase | 0.68 | 0 | secretory pathway |
| TGGT1_243730 | rhoptry protein ROP9 | 0.6 | 1 | secretory pathway |
| TGGT1_205250 | rhoptry protein ROP18 | 0.81 | 0 | secretory pathway |
| TGGT1_310010 | rhoptry neck protein RON1 | 0.98 | 1 | secretory pathway |
| TGGT1_315490 | rhoptry protein ROP10 | 0.95 | 0 | secretory pathway |
| TGGT1_258660 | rhoptry protein ROP6 | 0.77 | 2 | secretory pathway |
| TGGT1_247520 | hypothetical protein | 0.81 | 1 | secretory pathway |
| TGGT1_247960 | hypothetical protein | 0.42 | 1 | secretory pathway |
| TGGT1_201780 | microneme protein MIC2 | 0.97 | 1 | secretory pathway |
| TGGT1_297960A | rhoptry neck protein RON6 | 1 | 1 | secretory pathway |
| TGGT1_309590 | rhoptry protein ROP1 | 0.4 | 0 | secretory pathway |
| TGGT1_319560 | microneme protein MIC3 | 1 | 0 | secretory pathway |
| TGGT1_258580 | rhoptry protein ROP17 | 0.9 | 0 | secretory pathway |
| TGGT1_210370 | hypothetical protein | 0.99 | 0 | secretory pathway |
| TGGT1_261740 | hypothetical protein | 0.93 | 1 | secretory pathway |
| TGGT1_204050 | subtilisin SUB1 | 1 | 0 | secretory pathway |
| TGGT1_204530 | microneme protein MIC11 | 0.99 | 0 | secretory pathway |
| TGGT1_262730 | rhoptry protein ROP16 | 1 | 0 | secretory pathway |
| TGGT1_231640 | alveolin domain containing intermediate filament IMC1 | null | 0 | cytoplasmic |
| TGGT1_233820 | DNA polymerase epsilon subunit B protein | null | 0 | cytoplasmic |
| **TGGT1_230350** | **hypothetical protein (TAILS3)** | **null** | **6** | **cytoplasmic** |
| **TGGT1_239050** | **hypothetical protein (TAILS4)** | **null** | **0** | **cytoplasmic** |
| TGGT1_268835 | hypothetical protein | null | 0 | cytoplasmic |
| TGGT1_276190 | hypothetical protein | null | 7 | cytoplasmic |
| TGGT1_295360 | hypothetical protein | null | 0 | cytoplasmic |
| TGGT1_211330 | methionine aminopeptidase | null | 0 | cytoplasmic |
| TGGT1_273120 | SAG-related sequence SRS30C | null | 0 | (cytoplasmic) |
