# Supplementary material for: A druggable secretory protein maturase of Toxoplasma essential for invasion and egress
Source: eLife. 2017 Sep 12;6:e27480. doi: 10.7554/eLife.27480 (PMC5595437; doi:10.7554/eLife.27480)
Supplement: Supplementary file 4. — Predicted or experimentally validated subcellular localization is indicated. Gene IDs refer to ToxoDB, Release 31. [file elife-27480-supp4.docx]

**Supplementary File 4.** Eight proteins represented by peptides with normalized ATc+/ATc- abundance ratios of <0.22 and >2 (see also Figure 3C). Predicted or experimentally validated subcellular localization is indicated. Gene IDs refer to ToxoDB, Release 31.

| ToxoDB gene ID | Product | HMM signal peptide probability | # TM Domains | Trafficking |
| --- | --- | --- | --- | --- |
| TGGT1_261740 | hypothetical protein | 0.93 | 1 | secretory pathway |
| TGGT1_247520 | hypothetical protein | 0.81 | 1 | secretory pathway |
| TGGT1_204530 | microneme protein MIC11 | 0.99 | 0 | secretory pathway |
| TGGT1_319560 | microneme protein MIC3 | 1 | 0 | secretory pathway |
| TGGT1_291960 | rhoptry kinase family protein ROP40 (incomplete catalytic triad) | 0.83 | 0 | secretory pathway |
| TGGT1_262730 | rhoptry protein ROP16 | 1 | 0 | secretory pathway |
| TGGT1_243730 | rhoptry protein ROP9 | 0.6 | 1 | secretory pathway |
| TGGT1_204050 | subtilisin SUB1 | 1 | 0 | secretory pathway |
