# Supplementary material for: A druggable secretory protein maturase of Toxoplasma essential for invasion and egress
Source: eLife. 2017 Sep 12;6:e27480. doi: 10.7554/eLife.27480 (PMC5595437; doi:10.7554/eLife.27480)
Supplement: Supplementary file 6. [file elife-27480-supp6.docx]

**Supplementary File 6.** List of oligonucleotide primers used in this study.

| **Primer#** | **Gene/ Type** | **Primer Sequence** | **Descripton** |
| --- | --- | --- | --- |
| p30A | SAG1 | gtgacacctgcaagccacagcgg | Reverse primer in SAG1 3' UTR |
| 1245 | ASP3 | ggcactcgagcaaatccaagtccaaagtcttcgacc | Reverse primer in ASP3 |
| 1935 | SAG1 | cgctgcaccacttcattatttcttctgg | Sense 5’UTR of SAG1 |
| 2903 | tetR | gagcgagtttccttgtcgtcaggcc | Reverse primer in tetR region |
| 2794 | ASP3 | ccgggtacccctgcaggaccgaattcggcttaaattgccc | sense primer to amplify TgASP3 promoterKpnI-SbfI |
| M13f |  | tgtaaaacgacggccagt | M13 forward primer |
| 5987 | UPRT | ctggatgtgtcataccatggagtttcctgtaatacgactcactataggg | Forward primer universal to complement in the UPRT locus |
| 5988 | UPRT | actgcccgcaagccgctttccatcgactctcgggggggcaagaattgtg | Reverse primer universal to complement in the UPRT locus |
| 2797 | ASP3 | ccggagctccctgcaggcccctattgggcgaggatgc | Reverse primer in ASP3 3'UTR |
| 4938 | ASP3 | gtgcaagagatttctcacgaag | Forward primer to check intergration of ASP3 KI vector (outside of homology region) |
| 5000 | ASP3 | tcgtggtaccgaaagtgtttttacaaggtgtctgc | Forward primer with KpnI site to clone for ASP3 knock-in into the 3Ty vector pG152-KI-3Ty-lox-SAG1_3'UTR-HX |
| 5001 | ASP3 | gttatgcatccaattcacgcatcgacggcg | Reverse primer with NsiI site to clone for ASP3 knock-in into the 3Ty vector pG152-KI-3Ty-lox-SAG1_3'UTR-HX |
| 5038 | ASP3 | gggagcctgcccacggagacgggttcttcccgggtaccctcgggggggca | Forward primer for Tet inducible KD of ASP3 with CRISPR/Cas9 and TATi containing vector |
| 5039 | ASP3 | ggcccgccccgcggtagtccggccctccattttgtcgaaaaagggaattc | Reverse primer for Tet inducible KD of ASP3 with CRISPR/Cas9 and TATi containing vector |
| 5040 | ASP3 | gtttccccgttttctgagtctgttttagagctagaaatagc | guide RNA forward primer for Tet inducible KD of ASP3 with CRISPR/Cas9 and TATi containing vector |
| 5415 | ASP3 | ccatccatggtctagagtgtgttgccaacggaaaacc | Forward primer with NcoI site. Use with 5416 - PCR to amplify the 5' region of ASP3 (removing 230bp pre-ATG) and ligate it into 5'TgDGK1-pT8Tati1-HX-TetO7S1mycNtDGK1_NEW (plasmid1 from Silly Sausage) |
| 5416 | ASP3 | agacggatccatcaatacaggccg | Reverse Primer with BamHI site. Use with 5415 - PCR to amplify the 5' region of ASP3 (removing 230bp pre-ATG) and ligate it into 5'TgDGK1-pT8Tati1-HX-TetO7S1mycNtDGK1_NEW (plasmid1 from Silly Sausage) |
| 5417 | ASP3 | gcgcctagggatatcaaaatggagggccggactaccgcg | Forward primer with AvrII site. Use with 5418 - PCR to amplify the start region of ASP3 and ligate it into 5'TgDGK1-pT8Tati1-HX-TetO7S1mycNtDGK1_NEW (plasmid1 from Silly Sausage) |
| 5418 | ASP3 | gatgcggccgcgtacagtcacctgacatgagcag | Reverse primer with NotI site. Use with 5417 - PCR to amplify the start region of ASP3 and ligate it into 5'TgDGK1-pT8Tati1-HX-TetO7S1mycNtDGK1_NEW (plasmid1 from Hayley) |
| 5488 | ASP3 | gtcgagttcaccagtttcctca | Reverse primer to check for 3' integration of the ASP3/tet-inducible vector |
| 5735 | ASP3 | gccacgggcagcacgaatctctg | Forward primer for mutation of 'Asp' to 'Ala' in the pTub8-ASP3-Ty-HX vector (full length protein) |
| 5736 | ASP3 | gaagataggctggacgaactgag | Reverse primer for mutation of 'Asp' to 'Ala' in the pTub8-ASP3-Ty-HX vector (full length protein) |
| 5739 | MIC5 | ccggggccctgcgcctgaatgggatttttcg | Forward primer for MIC5 Knock-in ApaI site |
| 5740 | MIC5 | cgtatgcatatgcgagtttcacctcggagt | Reverse primer for MIC5 Knock-in NsiI site |
| 5741 | RON2 | ctgggcccgtacaacgcgtatctgcgaca | Forward primer for RON2 Knock-in ApaI site |
| 5742 | RON2 | ccgatgcataggctttgatgagaggcgcac | Reverse primer for RON2 Knock-in NsiI site |
| 5745 | RON5 | tatcagggcccgcctacaggtc | Forward Primer for RON5 Knock-in ApaI site |
| 5746 | RON5 | ggcctgcaggcagatccgggatttctttaaactg | Reverse Primer for RON5 Knock-in SbfI site |
| 5913 | SUB2 | gtcgggcccgtttggaccaaattaaagttcctg | Forward primer for SUB2 Knock-in - ApaI |
| 5914 | SUB2 | gtcctgcaggcgactcattctcgttgggcgt | Reverse primer for SUB2 KI - PstI |
| 5915 | SUB1 | ggatgacttctcatccgttagttttagagctagaaatagc | gRNA for inserting a tag before the GPI anchor site of TgSUB1 |
| 5916 | SUB1 | acgaaaatgacgcacgtgaagaggagccaccaaccgacgaggatgacttcgaggtccacacgaaccaggacccgctcgattcatccgttaagggtaaaaagttaggtgcctacgaatcggacggttcgcc | Forward Oligo for inserting a Ty tag (CRISPR-Cas9 mediated) before the GPI anchor site of TgSUB1 |
| 5917 | SUB1 | ggcgaaccgtccgattcgtaggcacctaactttttacccttaacggatgaatcgagcgggtcctggttcgtgtggacctcgaagtcatcctcgtcggttggtggctcctcttcacgtgcgtcattttcgt | Reverse Oligo for inserting a Ty tag (CRISPR-Cas9 mediated) before the GPI anchor site of TgSUB1 |
| 6283 | MIC6 | gatgaattcgagaatcgttactgtggctg | Forward primer for cloning MIC6 EGF domains with N-terminal GST |
| 6285 | MIC6 | gttactagtttaagcatgtccacttccttcctct | Reverse primer for cloning MIC6 EGF domains with N-terminal GST |
| 6435 | SUB2 | gcgaagaagtgacaggcaaagttttagagctagaaatagc | gRNA upstream of the start codon |
| 6532 | SUB2 | caacccagtggaaccgatttg | 5' forward check for recombination of the teti cassette |
| 6533 | SUB2 | tccgcaccagaggaaaaagtg | 3' reverse check for recombination of the teti cassette |
| 6625 | SUB2 | gagctatgttgtgtgtgagggttttagagctagaaatagc | gRNA2 for SUB2 KO |
| 6626 | SUB2 | gattggagtgatttcaacttctgtgtcagcggccgctctagaactag | Fwd primer for SUB2-KO (homology: 28 bp) with insertion of CAT cassette (19bp) |
| 6627 | SUB2 | tttgcttgtaaaaacatccctcccattcccctcgaggtcgacggtat | Rev primer for SUB2-KO (homology: 30 bp) with insertion of CAT cassette (19 bp) |
| 6634 | SUB2 | aggtcgacgacgtgagcagagc | Rev check primer for SUB2-KO |
| 6757 | TAILS1 | gtagggcccagaaactcgaccgagcgagac | forward primer ApaI for KI DHFR |
| 6758 | TAILS1 | gtacctgcaggggcgccggtaacctttacag | reverse primer with SbfI for KI DHFR |
| 6759 | TAILS3 | gtagggcccgaactgttcgaaggccgctac | forward primer with ApaI for KI DHFR |
| 6760 | TAILS3 | gtacctgcaggcactcgctctccaggaattgg | reverse primer with SbfI for KI DHFR |
| 6763 | TAILS7 | gtagggccccagagatccacgagcaatccc | forward primer with ApaI for KI DHFR |
| 6764 | TAILS7 | gtgcctgcaggatagccgattttcccgttttccg | reverse primer with SbfI for KI DHFR |
| 6765 | TAILS8 | gatgggccctcaccgacatcctcaaacagag | forward primer with ApaI for KI DHFR |
| 6766 | TAILS8 | acgcctgcagggctcacgacgattgttttatcg | reverse primer with SbfI for KI DHFR |
| 6769 | TAILS4 | gtagggcccgtcaaggaactccaggaactg | forward primer with ApaI for KI DHFR |
| 6770 | TAILS4 | gcgatgcatggactgtcgtggtgagagctg | Reverse primer with NsiI for KI DHFR |
| 6773 | TAILS6 | gtagggcccgtttggccaccgactgtc | forward primer with ApaI for KI DHFR |
| 6774 | TAILS6 | gcgatgcatggcttcgattctccacgtgtatttg | reverse primer with NsiI for KI DHFR |
| 6777 | TAILS2 | gtagggcccgaagaacctgcagcag | forward primer with ApaI for KI DHFR |
| 6778 | TAILS2 | gcgatgcatgcgcagagaccctggggctg | reverse primer with NsiI for KI DHFR |
| 6814 | TAILS1 | gtagggcccagcagcatccagagtctg | new forward primer with ApaI |
| 6873 | TAILS1 | actccgagctgaacggttc | forward primer integration 5' |
| 6874 | TAILS3 | tcttgaccgcgacgctgttc | forward primer integration 5' |
| 6875 | TAILS7 | gaggagatttcggtcagtgc | forward primer integration 5' |
| 6876 | TAILS8 | accggatacgtgtgaatggg | forward primer integration 5' |
| 6878 | TAILS4 | aattcctgtctggctgacg | forward primer integration 5' |
| 6880 | TAILS6 | agcggggagtataacttccg | forward primer integration 5' |
| 6882 | TAILS2 | gaatgcctgagaaagctgcag | forward primer integration 5' |
| 7092 | TAILS5 | tcgggcccgatgcttccattccttaacgatg | Forward primer with ApaI site for KI of TGGT1_258360 (TAILS5) to be cloned into the ApaI, NsiI digested KI-3Ty-DHFR vector |
| 7093 | TAILS5 | gctcctgcaggtcgctccagagagtgcaagc | Reverse primer with SbfI site for KI of TGGT1_258360 (TAILS5) to be cloned into the ApaI, NsiI digested KI-3Ty-DHFR vector |
| 7094 | TAILS5 | cttctcacccagcaactaaatg | Forward primer on the 5 side to check for integration of the KI vector |
| 7095 | TAILS5 | caacgacttgtaaacctttgtgg | Reverse primer on the 3 side to check for integration of the KI vector |
