# Supplementary material for: A druggable secretory protein maturase of Toxoplasma essential for invasion and egress
Source: eLife. 2017 Sep 12;6:e27480. doi: 10.7554/eLife.27480 (PMC5595437; doi:10.7554/eLife.27480)
Supplement: Supplementary file 7. [file elife-27480-supp7.docx]

**Supplementary File 7.** List of plasmids generated for this study

| **Plasmid** | **Description** |
| --- | --- |
| KI-ASP3ty-HX | For C-terminal epitope tagging (3Ty) of ASP3 (TGME49_246550) at endogenous locus |
| KI-ASP3ty-DHFR | For C-terminal epitope tagging (3Ty) of ASP3 (TGME49_246550) at endogenous locus |
| KI-ASP3-myc-Bleo | For C-terminal epitope tagging (myc) of ASP3 (TGME49_246550) at endogenous locus |
| pTub8-ASP3ty-HX | Plasmid with ASP3 cDNA |
| pTub8-asp3ty-D299A-HX | Plasmid with ASP3 cDNA with a Asp to Ala mutation in the first catalytic residue |
| 5’ASP3-pT8TATi1-HXtet07S1-ASP3 | For Recombination of the inducible cassette at the endogenous locus of RHΔku80 strain |
| 5'UPRT-pT8-ASP3ty -3'UPRT | For Second copy of 3Ty tagged wild-type ASP3 (TGME49_246550) in the UPRT locus |
| 5'UPRT-pT8-asp3ty-D299A -3'UPRT | For Second copy of 3Ty tagged catalytically dead mutant of ASP3 (TGME49_246550), with the Asparatate residue at 299 mutated to Alanine, in the UPRT locus |
| UPRT-Cas9-YFP/CRISPR | For integrating the second copy of WT ASP3 or the catalytically dead mutant in the UPRT locus |
| SUB1-Cas9-YFP/CRISPR | gRNA containing plasmid for Tagging (Ty) of SUB1 (TGME49_204050) before the GPI anchor addition site at the endogenous locus in the ASP3myc-iKD strain |
| SUB2-6435-Cas9-YFP/CRISPR | gRNA1 containing plasmid to generate the 2 guide RNA plasmid for SUB2 (TGME49_314500) knock-out |
| SUB2-6625-Cas9-YFP/CRISPR | gRNA2 containing plasmid to generate the 2 guide RNA plasmid for SUB2 (TGME49_314500) knock-out |
| SUB2-6434-6625-Cas9-YFP/CRISPR | Two guide RNA containing plasmid for generating SUB2 (TGME49_314500) konck-out, by replacing part of SUB2 locus with CAT cassette |
| KI-SUB2ty-DHFR | C-terminal epitope tagging (3Ty) of SUB2 (TGME49_314500) at endogenous locus |
| KI-ROP18ty-DHFR | C-terminal epitope tagging (3Ty) of ROP18 (TGME49_205250) at endogenous locus |
| KI-RON2ty-DHFR | C-terminal epitope tagging (3Ty) of RON2 (TGME49_300100) at endogenous locus |
| KI-RON5ty-DHFR | C-terminal epitope tagging (3Ty) of RON5 (TGME49_311470) at endogenous locus |
| KI-MIC5ty-DHFR | C-terminal epitope tagging (3Ty) of MIC5 (TGME49_277080) at endogenous locus |
| KI-TAILS1-3Ty-DHFR | C-terminal epitope tagging (3Ty) of TAILS1 (TGME49_202870) at endogenous locus |
| KI-TAILS2-3Ty-DHFR | C-terminal epitope tagging (3Ty) of TAILS2 (TGME49_225860) at endogenous locus |
| KI-TAILS3-3Ty-DHFR | C-terminal epitope tagging (3Ty) of TAILS3 (TGME49_230350) at endogenous locus |
| KI-TAILS4-3Ty-DHFR | C-terminal epitope tagging (3Ty) of TAILS4 (TGME49_239050) at endogenous locus |
| KI-TAILS5-3Ty-DHFR | C-terminal epitope tagging (3Ty) of TAILS5 (TGME49_258360) at endogenous locus |
| KI-TAILS6-3Ty-DHFR | C-terminal epitope tagging (3Ty) of TAILS6 (TGME49_273860) at endogenous locus |
| KI-TAILS7-3Ty-DHFR | C-terminal epitope tagging (3Ty) of TAILS7 (TGME49_279420) at endogenous locus |
| KI-TAILS8-3Ty-DHFR | C-terminal epitope tagging (3Ty) of TAILS8 (TGME49_321650) at endogenous locus |
