# Supplementary material for: A druggable secretory protein maturase of Toxoplasma essential for invasion and egress
Source: eLife. 2017 Sep 12;6:e27480. doi: 10.7554/eLife.27480 (PMC5595437; doi:10.7554/eLife.27480)
Supplement: Supplementary file 8. [file elife-27480-supp8.docx]

**Supplementary File 8.** List of strains generated for this study

| **Strain** | **Mode of generation** |
| --- | --- |
| RHΔku80 | WT strain used in the study |
| ASP3ty | C-terminal epitope tagging (3Ty) of ASP3 (TGME49_246550) at the endogenous locus |
| ASP3-iKD | Recombination of the pT8-Tati-HX vector at the endogenous locus of RHΔku80 strain |
| ASP3ty-iKD | C-terminal epitope tagging (3Ty) of ASP3 (TGME49_246550) at the endogenous locus of ASP3-iKD strain |
| ASP3myc-iKD | C-terminal epitope tagging (myc) of ASP3 (TGME49_246550) at the endogenous locus of ASP3-iKD strain |
| ASP3ty in ASP3myc-iKD | Second copy of 3Ty tagged wild-type ASP3 (TGME49_246550) in the UPRT locus |
| asp3ty-D299A in ASP3myc-iKD | Second copy of 3Ty tagged catalytically dead mutant of ASP3 (TGME49_246550), with the Asparatate residue at 299 mutated to Alanine, in the UPRT locus |
| ROP18ty/  ASP3myc-iKD | C-terminal epitope tagging (3Ty) of ROP18 (TGME49_205250) at the endogenous locus in the ASP3myc-iKD strain |
| RON2ty/  ASP3myc-iKD | C-terminal epitope tagging (3Ty) of RON2 (TGME49_300100) at the endogenous locus in the ASP3myc-iKD strain |
| RON4ty/  ASP3myc-iKD | C-terminal epitope tagging (3Ty) of RON4 (TGME49_229010) at the endogenous locus in the ASP3myc-iKD strain |
| RON5ty/  ASP3myc-iKD | C-terminal epitope tagging (3Ty) of RON5 (TGME49_311470) at the endogenous locus in the ASP3myc-iKD strain |
| SUB2ty/  ASP3myc-iKD | C-terminal epitope tagging (3Ty) of SUB2 (TGME49_314500) at the endogenous locus in the ASP3myc-iKD strain |
| MIC5ty/  ASP3myc-iKD | C-terminal epitope tagging (3Ty) of MIC5 (TGME49_277080) at the endogenous locus in the ASP3myc-iKD strain |
| SUB1ty/  ASP3myc-iKD | Tagging (Ty) of SUB1 (TGME49_204050) before the GPI anchor addition site at the endogenous locus in the ASP3myc-iKD strain |
| TAILS1ty/  ASP3myc-iKD | C-terminal epitope tagging (3Ty) of TAILS1 (TGME49_202870) at the endogenous locus in the ASP3myc-iKD strain |
| TAILS2ty/  ASP3myc-iKD | C-terminal epitope tagging (3Ty) of TAILS2 (TGME49_225860) at the endogenous locus in the ASP3myc-iKD strain |
| TAILS3ty/  ASP3myc-iKD | C-terminal epitope tagging (3Ty) of TAILS3 (TGME49_230350) at the endogenous locus in the ASP3myc-iKD strain |
| TAILS4ty/  ASP3myc-iKD | C-terminal epitope tagging (3Ty) of TAILS4 (TGME49_239050) at the endogenous locus in the ASP3myc-iKD strain |
| TAILS5ty/  ASP3myc-iKD | C-terminal epitope tagging (3Ty) of TAILS5 (TGME49_258360) at the endogenous locus in the ASP3myc-iKD strain |
| TAILS6ty/  ASP3myc-iKD | C-terminal epitope tagging (3Ty) of TAILS6 (TGME49_273860) at the endogenous locus in the ASP3myc-iKD strain |
| TAILS7ty/  ASP3myc-iKD | C-terminal epitope tagging (3Ty) of TAILS7 (TGME49_279420) at the endogenous locus in the ASP3myc-iKD strain |
| TAILS8ty/  ASP3myc-iKD | C-terminal epitope tagging (3Ty) of TAILS8 (TGME49_321650) at the endogenous locus in the ASP3myc-iKD strain |
| SUB2ty | C-terminal epitope tagging (3Ty) of SUB2 (TGME49_314500) at the endogenous locus of RHΔku80 strain |
| SUB2-KO | Deletion of SUB2 by recombination of CAT cassette in the SUB2 locus of the SUB2ty strain |
