# Supplementary material for: A druggable secretory protein maturase of Toxoplasma essential for invasion and egress
Source: eLife. 2017 Sep 12;6:e27480. doi: 10.7554/eLife.27480 (PMC5595437; doi:10.7554/eLife.27480)
Supplement: Supplementary file 9. [file elife-27480-supp9.docx]

**Supplementary File 9**. List of EuPathDB IDs of protein sequences used for phylogeny tree generation.

| **Gene ID** | **Organism** |  | **Gene ID** | **Organism** |
| --- | --- | --- | --- | --- |
| BBBOND_0107210 | *B. bigemina* strain BOND |  | PF3D7_1323500 | *P. falciparum* 3D7 |
| BBBOND_0108020 | *B. bigemina* strain BOND |  | PF3D7_1407800 | *P. falciparum* 3D7 |
| BBBOND_0302990 | *B. bigemina* strain BOND |  | PF3D7_1407900 | *P. falciparum* 3D7 |
| BBBOND_0311650 | *B. bigemina* strain BOND |  | PF3D7_1408000 | *P. falciparum* 3D7 |
| BBBOND_0400170 | *B. bigemina* strain BOND |  | PF3D7_1408100 | *P. falciparum* 3D7 |
| Cvel_10378 | *C. velia* CCMP2878 |  | PF3D7_1430200 | *P. falciparum* 3D7 |
| Cvel_11145 | *C. velia* CCMP2878 |  | PF3D7_1465700 | *P. falciparum* 3D7 |
| Cvel_17886 | *C. velia* CCMP2878 |  | PVX_085030 | *P. vivax* Sal-1 |
| Cvel_21827 | *C. velia* CCMP2878 |  | PVX_086040 | *P. vivax* Sal-1 |
| Cvel_6235 | *C. velia* CCMP2878 |  | PVX_088125 | *P. vivax* Sal-1 |
| Cvel_7851 | *C. velia* CCMP2878 |  | PVX_111035 | *P. vivax* Sal-1 |
| EAH_00057680 | *E. acervulina* Houghton |  | PVX_116695 | *P. vivax* Sal-1 |
| ETH_00007420 | *E. tenella* strain Houghton |  | PVX_117180 | *P. vivax* Sal-1 |
| ETH_00008525 | *E. tenella* strain Houghton |  | PVX_119690 | *P. vivax* Sal-1 |
| GNI_050090 | *G. niphandrodes* Unknown strain |  | SN3_00400750 | *S. neurona* SN3 |
| GNI_128160 | *G. niphandrodes* Unknown strain |  | SN3_00401155 | *S. neurona* SN3 |
| HHA_201840 | *H. hammondi* strain H.H.34 |  | SN3_00700635 | *S. neurona* SN3 |
| HHA_209620 | *H. hammondi* strain H.H.34 |  | TA02510 | *T. annulata* strain Ankara |
| HHA_242720 | *H. hammondi* strain H.H.34 |  | TA02750 | *T. annulata* strain Ankara |
| HHA_246550 | *H. hammondi* strain H.H.34 |  | TA05735 | *T. annulata* strain Ankara |
| HHA_261530 | *H. hammondi* strain H.H.34 |  | TA17685 | *T. annulata* strain Ankara |
| HHA_262940 | *H. hammondi* strain H.H.34 |  | TGME49_201840 | *T. gondii* ME49 |
| HHA_272510 | *H. hammondi* strain H.H.34 |  | TGME49_209620 | *T. gondii* ME49 |
| NCLIV_003910 | *N. caninum* Liverpool |  | TGME49_242720 | *T. gondii* ME49 |
| NCLIV_017720 | *N. caninum* Liverpool |  | TGME49_246550 | *T. gondii* ME49 |
| NCLIV_022920 | *N. caninum* Liverpool |  | TGME49_261530 | *T. gondii* ME49 |
| NCLIV_024980 | *N. caninum* Liverpool |  | TGME49_262940 | *T. gondii* ME49 |
| NCLIV_063340 | *N. caninum* Liverpool |  | TGME49_272510 | *T. gondii* ME49 |
| PBANKA_0409700 | *P. berghei* ANKA |  | TOT_010000649 | *T. orientalis* strain Shintoku |
| PBANKA_0517600 | *P. berghei* ANKA |  | TOT_010000759 | *T. orientalis* strain Shintoku |
| PBANKA_1014500 | *P. berghei* ANKA |  | TOT_030000196 | *T. orientalis* strain Shintoku |
| PBANKA_1034400 | *P. berghei* ANKA |  | TOT_030000542 | *T. orientalis* strain Shintoku |
| PBANKA_1222500 | *P. berghei* ANKA |  | TOT_030000809 | *T. orientalis* strain Shintoku |
| PBANKA_1329100 | *P. berghei* ANKA |  | cgd1_2240 | *C. parvum* Iowa II |
| PBANKA_1338700 | *P. berghei* ANKA |  | cgd1_3690 | *C. parvum* Iowa II |
| PF3D7_0311700 | *P. falciparum* 3D7 |  | cgd4_2190 | *C. parvum* Iowa II |
| PF3D7_0808200 | *P. falciparum* 3D7 |  | cgd6_3820 | *C. parvum* Iowa II |
| PF3D7_1033800 | *P. falciparum* 3D7 |  | cgd6_660 | *C. parvum* Iowa II |
