# Supplementary material for: A druggable secretory protein maturase of Toxoplasma essential for invasion and egress
Source: eLife. 2017 Sep 12;6:e27480. doi: 10.7554/eLife.27480 (PMC5595437; doi:10.7554/eLife.27480)
Supplement: Supplementary file 10. [file elife-27480-supp10.docx]

**Supplementary File 10**. Curated alignments used to generate the phylogenetic tree of Apicomplexan aspartyl proteases

10 20 30 40 50 60 70 80 90 100

....|....| ....|....| ....|....| ....|....| ....|....| ....|....| ....|....| ....|....| ....|....| ....|....|

cgd4_2190 **GYYFIKVNVG** **TQQQTLIIDT** **GSSLTGFACS** **DCGTHENKPF** **NINLSDTSNI** **IKCVYDIKYS** **EGSRLGYFFE** **DFVFSNKEMN** **GETDISIFFE** **KDGGKLTFGS**

Cvel_17886 **AYWFVDILVG** **PQRQSVIVDT** **GSTVLAYPCK** **NCGHHIDAPF** **DFSASETAQW** **QKCSYYQGYT** **EGSHMGYWFK** **DYVFSRNREH** **TSNTFALCLA** **DWGGVMTVGG**

cgd1_3690 **AYYYSDIFVG** **PQRQSVILDT** **GSNLLAFSST** **QCGTHLDAYY** **DPFKSITKRE** **VQCAYTIHYL** **EGSSSGSYFE** **DFVYTASSQE** **KYKDLSLCFS** **SEGGMISFGG**

PVX_116695 **AYYFLDIDIG** **EQRISLILDT** **GSSSLSFPCA** **GCGVHMENPF** **NLNNSKTSSI** **LKCEYMQSYC** **EGSQSGFYFS** **DVVLSKPQGI** **APLKFTICIS** **ENGGELIAGG**

PBANKA_133870 **AYYFMDINIG** **GQKLSLIVDT** **GSSSLSFPCS** **ECGVHMENPF** **NLNNSSTSSI** **LRCEYLQSYC** **EGSRNGFYFS** **DIVLTKPKGV** **SPLNFSLCIS** **EYGGELILGG**

PF3D7_1323500 **AYYFLDIDIG** **SQRISLILDT** **GSSSLSFPCN** **GCGIHMEKPY** **NLNYSKTSSI** **LKCEYLQSYC** **EGSQYGFYFS** **DIVLTKPNGV** **TPLKYSICVS** **EHGGELIIGG**

EAH_00057680 **AYYFADLFVG** **PQRQSLILDT** **GSSVMAFPCE** **HCNCAYRVSY** **MEGSSLQGFW** **FAAALGSPFL** **SGSSLDPPSF** **DTSVWNAFGP** **RFHHFALCLA** **EHGGVLTFGA**

ETH_00007420 **AYYFADLFVG** **PQRQSLILDT** **GSSVMAFPCE** **HCGKHIDEPF** **SCHKSPSCAY** **THCAYRVSYM** **EGSSQGFWFQ** **DEMVWNSFGP** **RFHHFAMCLS** **EHGGLLTFGG**

GjASP7 **AYYFSDVIFG** **SQRQSLILDT** **GSSVMGFPCS** **SCGDHIDPAF** **NCQKSSSCKP** **LKCAYRVAYM** **EGSAQGFWYH** **DSVLWSQFGP** **QSPPFSLCLS** **EHGGSFTIGN**

HHA_261530 **AYYFADVIVG** **VQRQSLILDT** **GSSVLAFPCT** **SCGRHMDPPF** **DCASSSTCES** **VRCAYRVSYM** **EGSSQGFWHE** **DQFIWSQFGP** **RAPDFALCLA** **EHGGAFSIGD**

TGME49_261530 **AYYFADVVVG** **VQRQSLILDT** **GSSVLAFPCT** **SCGRHMDPPF** **DCSSSSTCKS** **VRCAYRVSYM** **EGSSQGFWHE** **DQFIWSQFGP** **RAPDFALCLA** **EHGGAFSIGD**

Cvel_6235 **GYYFADILVG** **FQRQSVILDT** **GSAMTAFPCA** **SCGTVKDPFF** **NSSMSSTFYW** **HVCTYGVSYV** **EGSSAGKWFS** **DVASPRASSR** **SDDRLSICLA** **EFGGSMSVGG**

GjASP5 **AYYFLDVQVG** **PQRVSVILDT** **GSSLLAFPCV** **GCGIHVDRQF** **DPTISKTGEW** **VRCMYTQSYS** **EGSSSGIYFS** **DLVFPKGFRQ** **EVDQFSVCIS** **DDGGLLTVGG**

SN3_00401155 **AYYFLDILVG** **PQRASVILDT** **GSSLLAFPCK** **GCGEHLDPAI** **DTGLTTTGEW** **VRCMYSQTYS** **EGSSRGIYMS** **DVVFPKGHRQ** **ELDRFSICIS** **EDGGLLTVGG**

NCLIV_017720 **AYYFLDILVG** **PQRASVILDT** **GSSLLAFPCA** **GCGEHLDPAM** **DTSRSATGEW** **IRCMYTQTYS** **EGSARGIYFS** **DVVFPKGHRQ** **NLAQFSVCIS** **EDGGLLTVGG**

HHA_242720 **AYYFLDILVG** **PQRASVILDT** **GSSLLAFPCA** **GCGQHLDPAM** **DTSRSATGEW** **IRCMYTQTYS** **EGSARGIYFS** **DVVFPKGHRQ** **DLDKFSVCIS** **EDGGLLTVGG**

TGME49_242720 **AYYFLDILVG** **PQRASVILDT** **GSSLLAFPCA** **GCGQHLDPAM** **DTSRSATGEW** **IRCMYTQTYS** **EGSARGIYFS** **DVVFPKGHRQ** **NLDKFSVCIS** **EDGGLLTVGG**

BBBOND_0302990 **AYYYTVVEVG** **PQSQEVVVDT** **GSANLVLADS** **ECGHHDMKPF** **NTTLSRTLSY** **IAIKDACVFA** **ESAISGFYAT** **DYFVSSDDSS** **NFTRFELCLS** **EDGGSLVFGG**

BBBOND_0107210 **ATYYGEIILG** **GDTFKVLFDT** **GSSELWVPDE** **LCACLTRKRL** **SRAERWTAKY** **DYIPILVKYL** **TGEMRAIDGT** **ADVFTTDDQT** **AVPHFAYYIS** **KTGGNVTFGG**

TA05735 **ATYYGNIILG** **RNSFKVLFDT** **GSSEFWVPYE** **MCQCLNRKRY** **HKGSEWIAKH** **DYVPLEIKYL** **SGQIDAIDGT** **ATVFKTKDME** **KSPNVAYYVT** **KNGGNISFGG**

TOT_010000759 **ATYYGNIIVG** **TNSFRVLFDT** **GSSEFWVPYE** **LCQCLSRKRY** **HKTYEWRAKR** **DYVPIEIKYL** **SGEIDAIDGT** **TNVFKTKEME** **KSPNVAYYVS** **KNGGNISFGG**

PVX_111035 **TTYYGQVAVG** **ENVVNVLFDT** **GSTEFWIPFE** **NCNFPDHKKY** **KRTKSFRNKF** **NPTLLEVNYL** **SGKVIGFDGY** **DTVFENEDSR** **NIRGFGYYLS** **DRGGFISFGG**

PBANKA_051760 **STYYGKIAIG** **ENIFNVLFDT** **GSTEFWVPFK** **TCKNNIHNKY** **ERTQSFKYKY** **DPSVLEINYL** **SGKLVGFDGY** **DTVFENEDSQ** **KIKNFGYYIT** **NTGGYITLGG**

PF3D7_1033800 **STYYGEVQIG** **ENNMNVLFDT** **GSSQVWILND** **TCLCNNHSKY** **KRTKSFVYKY** **DPSVIEIFYL** **SGKIVAFEGY** **DTIFQNGDSI** **KIKNFGYYLS** **DKEGYITLGG**

GjASP6 **ATYFADIGLG** **TTNFKVLFDS** **GSCEFWVPDE** **ECQCMGHTKY** **RKSSTFEPRF** **TPSLMDVVYL** **SGTLQGYDGY** **ETVVRQTDFL** **NVNAFAYYIN** **SLGGSVTFGG**

HHA_272510 **ATYYGEISIG** **ERAFRVLFDT** **GSCEFWVPDE** **TCQCIGHRKY** **RRSASFQPRF** **NPSLMNVVYL** **SGTLQGYDGY** **DTVFKNHEIS** **KAKNFAYYLN** **ADGGSMTFGG**

TGME49_272510 **ATYYGEISIG** **ERAFKVLFDT** **GSCEFWVPDE** **TCQCIGHTKY** **RRSASFQPRF** **NPSLMNVVYL** **SGTLQGYDGY** **DTVFKNHEIS** **KAKHFAYYLN** **ADGGSMTFGG**

NCLIV_003910 **TQYFGEISVG** **PSLFKVVFDS** **GSHQFWIPSK** **ECSCRTHSRL** **DCSRSSSCRD** **HNESVAVTFG** **TGRIVYRKAL** **ETVLLSGSRL** **GITDFAFYLS** **RRSGVISFGG**

HHA_209620 **TQYFGKISVG** **PQSFNVVFDT** **GSHHFWIPSK** **ECSCRAHCRF** **DGGRSSSFRH** **HNECVTVTFG** **TGRVVYRKAL** **EAILSRSSCV** **RVADFAVYLS** **RRRGVISFGG**

TGME49_209620 **TQYFGKISVG** **PQSFNVVFDT** **GSHHFWIPSN** **ECSCRAHSRF** **DDSRSSSFRH** **HNECVTVTFG** **TGRVVYRKAL** **EAILSRSSCL** **RVADFAVYLS** **RRRGVISFGG**

Cvel_7851 **TEYFGKISVG** **PKEFTVVFDT** **GSGNLLIPSI** **DCACSERPLY** **DPGNSSTSRQ** **IRDVITITFG** **TGEISGVVVR** **DTVLDGMSEG** **GHEHFSVFLG** **DGESEITFGN**

Cvel_11145 **SEYYGRITIG** **PQEFLVVFDT** **GSGNLLIPST** **DCACTSHKRY** **DAGTSKSAKQ** **VRDVVTITFG** **TGEMTGVFVR** **DRVLKEMSEG** **RVKRFAVFFG** **GSDSEITFGE**

PBANKA_132910 **RQFIGEISIG** **PQSFKVLFDT** **GSTNLWIPSK** **NCACYNKKKY** **DYNISKNYRI** **SKNPVNIFFG** **TGKVQIAYAT** **DDIISEDIKR** **NSRKFSIYYP** **KSVGAITFGG**

PF3D7_1465700 **RQFIGEINIG** **PQTFKVLFDT** **GSTNLWIPSK** **NCACYNKRKY** **DHKISKNYKL** **VKDPVEILFG** **TGEIHIAYVT** **DDIISDDKKK** **NIEKFAIYYP** **KNVGAITFGG**

PVX_117180 **RQFIGEIRIG** **PQAFKVLFDT** **GSTNLWIPSK** **NCACQSKRKY** **DHRVSKNYKS** **VKNPVEVFFG** **TGKIQIAYVS** **DDVISDDKRR** **GRKKFSIYYP** **KNVGAITFGG**

GNI_050090 **LIFIGEISVG** **PQPFTVVFDT** **GSSFFWLPSS** **DCGCEPHSKF** **DARRSQSYEA** **DSDVNFIQYG** **TGSCLLEFGR** **DRVYASEGSR** **QVKKFDFRAR** **NEGGWMYLGS**

cgd6_3820 **SQYFGKIEVG** **PREFVVIFDT** **GSSSVWIPSI** **ECGCEPHNKY** **DPKLSTSYQK** **LSLETYIQYG** **TGSCVLKFGK** **EVIFPDKNSK** **NIPRFGVYIS** **RDSGSISFGA**

BBBOND_0311650 **NQYFGEIEVG** **PSKFVVVFDT** **GSSQLWIPSK** **QCGCARHRQF** **DSTQSSTYRE** **PASNAYIQYG** **TGECVLALGA** **DTVFPDAAFS** **KLNRMAFYMT** **KDRGTLSFGS**

TA02510 **SQYFGEIQVG** **PKNFVVVFDT** **GSSQLWIPSK** **SCGCARHRMF** **DSSASTTYEP** **MMMSEYIRYG** **TGECVLALGF** **DNVFPDTELK** **KIKRIAFYMS** **KDIGSLSFGS**

TOT_030000809 **SQYFGEIEIG** **PKSFVVVFDT** **GSSQLWVPSK** **LCGCNRHRLF** **DFSKSSTYQP** **MILSEYIRYG** **TGECVLTLGF** **DNVFPDSELN** **KVKRIAFYMS** **KDVGLLSFGS**

NCLIV_024980 **SQYFGEIQVG** **PVPFVVVFDT** **GSSNLWIPAS** **ECGCMPHTRF** **DPKTSSTYLP** **IPAIAFIQYG** **TGACVLRMAK** **DTVFPDVTGE** **KLKRFAVYMS** **EQLGEITFGS**

HHA_262940 **SQYFGEIQVG** **PVSFIVVFDT** **GSSNLWIPAS** **ECGCVPHTRF** **DPKTSSTYLP** **IPAIAFIQYG** **TGACVLRMAK** **DTVFPDVAGE** **KLKRFAVYMS** **EDLGEITFGS**

TGME49_262940 **SQYFGEIQVG** **PVSFIVVFDT** **GSSNLWIPAS** **ECGCVPHTRF** **DPKTSSTYLP** **IPAIAFIQYG** **TGACVLRMAK** **DTVFPDVAGE** **KLKRFAVYMS** **EDLGEITFGS**

PVX_119690 **SQFIGDIQIG** **PQSFRVVFDT** **GSSNFALPST** **KCGCASHKKF** **NPDESRTYAR** **QSIYTYIQYG** **TGRSILEHGY** **DDVFSDPDFS** **NLKRFSFYVP** **KKLGSITFGR**

PBANKA_040970 **SQFIGDIEIG** **PQSFKVVFDT** **GSSNFAIPST** **KCGCTLHNKF** **DAKKSRTFMS** **NSIYTYVQYG** **TGKSILEHGY** **DDVFSDPDNS** **NLQQFSFYVP** **KELGAITFGR**

PF3D7_0311700 **SQFIADIGVG** **PQVFKVVFDT** **GSSNLAIPST** **KCGCASHKKF** **NPNKSRTFTK** **NSVYTYIQYG** **TGTSILEQSY** **DDVFSDPDFR** **NLKRFSFYVP** **KKLGAITFGK**

GNI_128160 **TQYYGVVGIG** **PQNLKVIFDT** **GSSNLWVPDS** **DCTCLGHDTF** **DPDKSNTYLP** **TDKKFEIKYG** **SGSVKGELAN** **DSVWSSISVG** **GLKDFAFHLG** **TRDGTLSIGG**

PBANKA_103440 **LSFIGTAELG** **KQSFSFILDT** **GSANLWVPSK** **ECGCAYKHRY** **DSSASNTYEK** **DGTPVSILYG** **SGGIKGFFSN** **DMFWKSLAVG** **GQENFSFYLP** **EADGYFTIGG**

PF3D7_1408100 **VLSFGEAKLG** **GQKFNFLFHT** **ASSNVWVPSI** **KCSCESKNHY** **DSSKSKTYEK** **DDTPVKLTSK** **AGTISGIFSK** **DLVWKDLSIG** **NKEQYSIYLP** **PENGYLTIGG**

PF3D7_1407800 **LMFYGEGQIG** **KQPFMFIFDT** **GSANLWVPSV** **NCGCSTKHLY** **DASASKSYEK** **DGTKVEISYG** **SGTVRGYFSK** **DVIWKDLSIG** **NKDNFTFYLP** **VHDGYLTIGG**

PVX_086040 **IMFYGEGEVG** **HQKFMLIFDT** **GSANLWVPSK** **KCGCSIKNLY** **DSSKSKSYEK** **DGTKVDITYG** **SGTVKGFFSK** **DLVWKDLSIG** **NKDNFTFYLP** **VHDGYLTIGG**

PF3D7_1408000 **IMFYGDAEVG** **QQPFTFILDT** **GSANLWVPSV** **KCGCLTKHLY** **DSSKSRTYEK** **DGTKVEMNYV** **SGTVSGFFSK** **DLVWKDLSIG** **NKENFTFYLP** **VHDGFLTIGG**

PF3D7_1407900 **VMYYGEAQIG** **KQKFAFIFDT** **GSANLWVPSA** **QCGCKTKNLY** **DSNKSKTYEK** **DGTKVEMNYV** **SGTVSGFFSK** **DIVWKDLSIG** **NKEQFTFYLP** **FDDGYLTIGG**

SN3_00700635 **SQYVIELGVG** **LQTVRAIADT** **GSSDFWACSE** **KCFCMLHKVY** **NHGKSSTYEP** **DNSPYNITYV** **GGPVSGFLSV** **DNVLPATSTY** **QLKKFAFYLS** **SDDGELTLGG**

NCLIV_022920 **AQYYTEIYVG** **GQKVRVVVDT** **GSSDLWVCSA** **SCLCMLHKTY** **NHGKSETYQE** **DGTPYHVEYA** **SGPVGGFLSV** **DDVFPALATN** **NVKNFAFYLG** **SANGELAIGG**

HHA_201840 **SQYYTEIYVG** **GQKVRVVVDT** **GSSDLWVCSA** **SCLCMMHKTY** **NHGKSETYHA** **DGTPYHVQYA** **SGPVGGFLSA** **DDVFPSLATK** **NVKNFAFYLA** **SANGELAIGG**

TGME49_201840 **SQYYTEIYVG** **GQKVRVVVDT** **GSSDLWVCSA** **SCLCMMHKTY** **NHGKSDTYHA** **DGTPYHVQYA** **SGPVGGFLSA** **DDVFPSLATK** **NVKNFAFYLA** **SANGELAIGG**

Cvel_21827 **AQYYGPITVG** **GQEFQVIFDT** **GSSNLWIPSK** **ECGCGTHALY** **DHSASKEYSE** **DGRKFHIEYG** **SGPVDGYLST** **DTVFPSIAVE** **GLKEFSFFLG** **KEDGELVFGG**

Cvel_10378 **AEYYAQVQVG** **KQTFNLILDT** **GSANVWVPAK** **GGDPLKHTLY** **DNTLSSSYVS** **LLMPFNIMYG** **SGPVSGKMAS** **DTMFAAISEG** **GLPELSFWMS** **SKESEVVFGG**

BBBOND_0108020 **TVLVIEASIG** **EQRFLPMLDT** **GSTNVWVVHE** **ACGCRGSMKY** **DPRKSSTFHS** **AKSYVRAKFV** **SGEVLGELGF** **EDFFPDLMIV** **LDAAFSFYYS** **LDGSAVLLGG**

cgd6_660 **SLFVCRIRIG** **EQEFWPIIDT** **GSSNLWVIGE** **ECSCQKVKRY** **SKYISKSFKR** **IYDNISVIFG** **TGKIYGKLIL** **ETLFTQMSSS** **GLQNFSIYIN** **DINAILLLGG**

TA02750 **VQYALNMGVG** **KQEINPIIDT** **GSTNTWVISQ** **NCTCEGVASF** **NSKKSETFNP** **INEGLKIKFG** **TGIIKGVLGI** **DNIFPKLAFD** **QIADLIFYFS** **NQYSYLMMGG**

TOT_010000649 **VQYALSIDVG** **PQSIYPIIDT** **GSTNTWVVSR** **KCTCKSVKSF** **DSSLSSTFGS** **LGDDIRIRFG** **TGVIRGSLGL** **DNVFPRLAFD** **KIADLTFYFS** **DNNSGLMIGG**

PBANKA_122250 **SQFVGKLLVG** **PQEIHPIFDT** **GSTNLWVVTT** **ECSCKKVHQY** **NPNKSKTFRR** **SKQNLHIVFG** **SGSITGTLGK** **DNFFPGMLTA** **YKMTFSFYIS** **PNDSTFIVGG**

PF3D7_0808200 **SQFVGELLVG** **PQTVYPIFDT** **GSTNVWVVTT** **ACSCKKVRRY** **DPNKSKTFRR** **SEKNLHIVFG** **SGSISGSVGT** **DTFFPGMLSA** **NPVDFSFYIS** **PYDSTLIIGG**

PVX_088125 **SQFVGTLLVG** **PQEIHPIFDT** **GSTNLWVVTT** **DCSCKKVKRY** **NPYKSKTFRR** **SGKNLHIVFG** **SGSISGSIGK** **ETFFPEMLSA** **NKLSFSFYIS** **PEDSTFLVGG**

PVX_085030 **SQYVGYIQIG** **PQTIRPIFDT** **GSTNIWVVST** **KCTCLKVHRY** **NYKLSRSFRY** **YHTNLDIMFG** **TGIIQGVIGV** **ETFFPAMLST** **YKSHFSIYIG** **KDNSALIFGG**

PBANKA_101450 **SQYVGSIQIG** **PQTIRPIFDT** **GSTNIWVVST** **KCTCLKVHRY** **NHKLSNTFKY** **YRTNLDIMFG** **TGIIQGTIGI** **DTFFSTMLST** **YNPHFSIYIG** **MDNSALIFGG**

PF3D7_1430200 **SQYVGYIQIG** **PQTIRPIFDT** **GSTNIWIVST** **KCTCLKVHRY** **NHKLSSSFKY** **YHTNLDIMFG** **TGIIQGVIGV** **ETFFPEMLST** **YKQHFSIYIG** **KDSSALIFGG**

TOT_030000542 **SLYVGSMYVG** **PQLMHPIFDT** **GSTNMWVVGT** **NCTCKKVKRF** **NPSLSNTFKA** **LPKRIHIRFG** **TGEIEGKPAK** **DVVFSEMSSI** **NLSHFSFYIG** **EDTALLMFGG**

BBBOND_0400170 **SLYVGEIKIG** **PQTFHPIFDT** **GSTNLWVVGS** **TCSCSKVCRY** **DASRSTTFKM** **MPIKIHIKFG** **TGEIEGYPAK** **DTVFPEMSSV** **KNKHFAFYID** **ENDSRIMLGG**

GjASP3 **SQYVGILGIG** **PQYVSPIFDT** **GSTNLWVVGS** **NCTCTKVTRF** **APDKSSTFHF** **RKVHLDITFG** **TGKIEGSTAI** **DNFFPDMSST** **GGKKFAFYVA** **DRSSALFFGG**

SN3_00400750 **SQYVGLLGIG** **PQYVQPVFDT** **GSTNLWVVGS** **DCTCEKVTRF** **DPRASSTYRP** **SPVNLDITFG** **TGRINGCTAV** **DDFFPEMSST** **GKKAFAFYMA** **KGFSGLFFGG**

ETH_00008525 **SQYCGMLGIG** **PQWVHPIFDT** **GSTNLWVVGS** **KCTCTKVTRF** **DPSKSSSFQY** **TPVHLDITFG** **TGRIEGSTGV** **DNFFPDMSST** **ADKRFAFYVA** **KDSSALFFGG**

NCLIV_063340 **SQYVGVIGIG** **PQFVKPIFDT** **GSTNLWVVGS** **KCTCTKVTQF** **DPSASSTFRT** **APVHLDITFG** **TGRIEGSTGI** **DDFFPEMSST** **GTKEFAFYMA** **KGSSALFFGG**

HHA_246550 **SQYVGVIGIG** **PQFVQPIFDT** **GSTNLWVVGS** **KCTCTKVTRF** **DPSASKTFRA** **TPVHLDITFG** **TGRIEGSTGI** **DDFFPEMSST** **GTKEFAFYMA** **KGSSALFFGG**

TGME49_246550 **SQYVGVIGIG** **PQFVQPIFDT** **GSTNLWVVGS** **KCTCTKVTRF** **DPSASKTFRA** **APVHLDITFG** **TGRIEGSTGI** **DDFFPEMSST** **GTKEFAFYMA** **KGSSALFFGG**

cgd1_2240 **GYYFINAFVG** **PQKQTLILDS** **GSSQISFTCI** **TCGSHEYPPF** **DIMKSITGKN** **CKCKYFHRFN** **EGSVSGKYFS** **DTLLKSSLDD** **TNKGISICLL** **YSGGRIVIGE**

TA17685 **AFYYIYMGIG** **KVKQMLIIDT** **GSQQINVACG** **NSGKHSLDNY** **NYQNSVTYKP** **ISCIFSETYS** **EGSNKGMYIG** **DLVRSDKNPL** **HFRHFSLCLS** **EDGGVLTLGG**

TOT_030000196 **AYYYVYVGIG** **KTKQMLIIDT** **GSQLINVACG** **KCGNHLLPNY** **ELGASVTHKL** **ISCLFNESYS** **EGSNEGKVVG** **DLIKSDKPTL** **HFMKFSLCLS** **ENGGVMTLGG**

110 120 130 140 150 160

....|....| ....|....| ....|....| ....|....| ....|....| ....|....| ....

cgd4_2190 **TCPFENYNIT** **RCINDERYCA** **YISEVDTKLN** **AIFDTGTTIS** **IFPARFKKIT** **RLFNTCWRML** **NIKV**

Cvel_17886 **SNKDPEILWT** **PLKDHTYYTV** **NLMSIGGGPT** **TIVDSGTTYT** **YLPPSWRLLK** **SSLDTCWRLS** **NITA**

cgd1_3690 **YNLDSKIGWT** **PLSILNNYYV** **QLTSVYHKDS** **LVIDSGTTLS** **YFPEHFIQIL** **NVINRCWKLR** **TITL**

PVX_116695 **YDEAEKVVWE** **NVTRKYYYYI** **KVRDMFMSSS** **MLVDSGSTFT** **HIPEDYNKLN** **YFFDQCWKYL** **DLFV**

PBANKA_133870 **YSIVDGILWE** **AITRKYYYYI** **RVKQLFSHNN** **MLVDSGSTFT** **HLPDDYNNLN** **FFFDQCWRYL** **NIYI**

PF3D7_1323500 **YENVEDIVWQ** **AITRKYYYYI** **KIYDLYMDKK** **MLVDSGSTFT** **HIPENYNQIN** **YYLDQCWKSL** **NLYI**

EAH_00057680 **ANHQQPPNWT** **SIIGIRSYNV** **RLAKVGTAAA** **VLIDSGSTLA** **YFPRHYKQIV** **AAIEAAGAAA** **RLLQ**

ETH_00007420 **AHHLQPPQWT** **PIIGMRSYNV** **RLARIGAAAA** **VLIDSGSTLA** **YFPRPYKQIV** **TAIEPSDGAS** **RLLQ**

GjASP7 **RNPSASSPFL** **PIIPHQTYSV** **FLTEIEEKRE** **MLIDSGTTLS** **YFPPHYNVIV** **QKIDGCNRDG** **SMAG**

HHA_261530 **ANHTSDTVAT** **NFIPHQSYSA** **YLSSVGERTN** **VLLDSGTTMS** **YFPTRYDEIV** **SAIESAWSAE** **DLTT**

TGME49_261530 **ANHTSDTVTT** **NFIPHQSYSA** **YLSSVGERTD** **VLLDSGTTMS** **YFPTRYDEIV** **SAIESAWSAE** **DLTT**

Cvel_6235 **FNSGPGRNWT** **PLIHPRRYYV** **RSTKGPADMG** **LLLDSGTTYS** **YLRRSYTRFR** **DLVNLCYDLP** **KVTL**

GjASP5 **FEADFPLIWA** **SIISHSAYRI** **PLRIVNSSAE** **TVVDSGTTYS** **YLPPAFARWR** **NIIDPCWRMN** **SLYL**

SN3_00401155 **YEYSDLIGWT** **PIISHSTYRV** **LLEEIEHDRE** **TMVDSGTTYS** **YFPPAFARWR** **QFLHLCWRVS** **PIKV**

NCLIV_017720 **YEHASLLTWT** **SIISHSTYRV** **PLSEVENGVD** **TMVDSGTTYS** **YFPPAFARWR** **SFLSPCWRVS** **PIKV**

HHA_242720 **YEHATLLTWT** **SIISHSTYRV** **PLSEVDSGVD** **TMVDSGTTYS** **YFPPAFSRWR** **SFLSPCWRVS** **PIKV**

TGME49_242720 **YEHAALLTWT** **SIISHSTYRV** **PLSEVESGVD** **TMVDSGTTYS** **YFPPAFSRWR** **SFLSPCWRVS** **PIKV**

BBBOND_0302990 **LEVDGPLLWM** **PLMRRGAYAI** **VVNTFCQPAN** **FILDSGSTNS** **SLEQPYSVIY** **GYMSLCNSMN** **TTRR**

BBBOND_0107210 **YNPADEFQWA** **PVSNKGYWAV** **NLLTVKNQPN** **VIIDTGTYLI** **YAPQNQNLVS** **SFVDSCDAKR** **TLVF**

TA05735 **INLDDEFLWA** **PVVKDSYWTL** **KLKKIKVKQY** **VIMDTGSFLI** **YAPQSMGPLL** **SRLQSCKEMN** **SLIF**

TOT_010000759 **INQNDEFVWA** **PIVKDSYWSL** **KLKKFKSANS** **VIMDTGSFLI** **YAPQSMSPLL** **AKLQSCDEVM** **VLVF**

PVX_111035 **VDSEEEVMWS** **PVSTEMFWTI** **DIVRKEVKGN** **SIVDTGTFFI** **YAPKKMESYL** **SGLESCEDKD** **YIVF**

PBANKA_051760 **IDPDEKIIWS** **PVSTEMFWTI** **DILRKEMNER** **SIVDTGTFLI** **YAPKKIENYL** **NDLTSCEDKQ** **YIIF**

PF3D7_1033800 **IDPDEEIIWT** **PVSTEMYWTI** **QIMRKEEENK** **SIIDTGTYLI** **YAPKNMENYL** **KDLKNCDEKY** **HLIF**

GjASP6 **VDANEEFSWV** **PVDPRNYWTV** **KVLRKEIPRS** **SIVDTGTYLI** **YAPSTMYDYL** **GDVRSCADRK** **DLVF**

HHA_272510 **ADPTEEFSWI** **PVDPSDYWTV** **KVIHKEKQLE** **SIVDTGTYLI** **YAPATMENEL** **RDISSCDDKK** **DLIL**

TGME49_272510 **ADPTEEFSWI** **PVDPSDYWTV** **KVIHKEKQLE** **SIVDTGTYLI** **YAPATMENEL** **SDISSCDDKK** **DLIL**

NCLIV_003910 **FDPGKPIQWI** **GMLPQKGWAM** **PLIKVDCFDS** **AVLDTGTSSI** **GGPRAIHHIL** **TMLGRCERKL** **SLTV**

HHA_209620 **FDQGKNIQWF** **ATLPQKGWAI** **PLIKVDCFDS** **AVLDTGTSSI** **GGPKAIHHVL** **TALGSCERRV** **HLTV**

TGME49_209620 **FDQGKNIQWF** **ATLPQEGWAI** **PLIKVDCFDS** **AVLDTGTSSI** **GGPKAIHQVL** **TALGSCERRV** **HLTV**

Cvel_7851 **WRMDSDLVWI** **PVSKEGYWQV** **TMQSIGCKIA** **VAVDTGTSLM** **AGPSDVAELS** **ARLNDCSNFD** **DLGF**

Cvel_11145 **WKMSSELFWV** **PVSHPGYWQV** **GMQVIGCGGH** **VAVDTGTSLM** **AGPTEIDALV** **ETLDDCSNFD** **DLGF**

PBANKA_132910 **YDPNSNIDWF** **VVSSRKYWTI** **KMTKINCSGN** **AVIDTGTSSI** **AGPQNLILLT** **KLLNSCQNKT** **NFSF**

PF3D7_1465700 **YDENSSIEWF** **DVTSSKYWAI** **QMKKINCSKN** **AVIDTGTSSI** **AGPKELILLS** **RLLNFCQKRI** **NFSF**

PVX_117180 **YDPNEKIDWF** **DVSSRKYWAV** **KMIKINCSKN** **AVIDTGTSSI** **AGPKDLILLT** **RLLNSCHNRA** **RFSF**

GNI_050090 **FPHTRDWMWF** **PLSSLRHWEV** **PLSKVNCATP** **AAIDTGSSLV** **TVPSRFMHLV** **HALNPCHSLH** **EISF**

cgd6_3820 **ADSGQKITWH** **RLTGSHYWEI** **KIKKINCFGD** **AAIDTGSSVS** **TAPSSMRKIT** **KVIPECNRYL** **RITY**

BBBOND_0311650 **IDPGHSPWWF** **PVVSTDFWEI** **EMDLIDELQR** **AAIDTGSSLI** **SGPSEVGPLL** **EKLSDCSNAA** **TISF**

TA02510 **IDPGHKPWWF** **PVVKTDYWEI** **EVSLVDVFDR** **AAIDTGSSLI** **SGPSDIIPLL** **EKITDCSNLD** **RLSF**

TOT_030000809 **IDPGHKPWWF** **PVVKTDYWEI** **EVDFVDPFDK** **AAVDTGSSLI** **SGPSEVIPLL** **EKIDDCSNIK** **RISF**

NCLIV_024980 **VNEGHKPQWF** **PVISLDYWEV** **GIHRLNCERK** **AAVDTGSSLI** **TGPSEINPLL** **QSLNDCSNKD** **TVTF**

HHA_262940 **VNEGHKPQWF** **PVISLDYWEV** **GVHRLNCERT** **AAVDTGSSLI** **TGPSKINPLL** **KSLNDCSNKG** **TVTF**

TGME49_262940 **VNEGHKPQWF** **PVISLDYWEV** **GVHRLNCERT** **AAVDTGSSLI** **TGPSKINPLL** **KSLNDCSNKG** **TVTF**

PVX_119690 **ANEGRQVEWF** **PVISIYFWEI** **NLIQLSCENR** **AAIDTGSSLL** **TGPSSMQPLI** **EKLDDCSNKS** **NISF**

PBANKA_040970 **ANEGEKIEWF** **PVISMYFWEI** **NLLLLPCSNK** **AAVDTGSSLI** **TGPSSMQPLI** **ENINDCSNIS** **IISF**

PF3D7_0311700 **ANEGKSIEWF** **PVISLYYWEI** **NLLQLSCESK** **AAIDTGSSLI** **TGPSTIQPLL** **EKINDCSNKE** **IISF**

GNI_128160 **YKDNGAITWI** **PLASKDYWTI** **RLEQMASRSD** **AIVDSGTSLI** **AAPISVEKIS** **RLIGSCDKKV** **NLDF**

PBANKA_103440 **IEYTGDVTYE** **KLTNESYWQI** **NLDDFGALNN** **AIVDSGTSAI** **TAPSDLNKFL** **NSIMLCHDTN** **TLKF**

PF3D7_1408100 **IEFDGPLNYE** **KLNHDLMWQV** **DLDHFGSSKK** **VILDSATSVI** **TVPTEFNQFV** **ESASTCGNTK** **TLEY**

PF3D7_1407800 **IEYEGPLTYE** **KLNHDLYWQI** **DLDHFGQKAN** **AVVDSGTSTI** **TAPTSLNKFF** **RDMNTCDNDD** **TLEF**

PVX_086040 **IEYEGNITYE** **KLNHDLYWQI** **DLDHFGEKAN** **VIVDSGTTTI** **TAPSELNKFF** **ANLNTCDNKE** **TLEF**

PF3D7_1408000 **IEYEGPLTYE** **KLNHDLYWQI** **TLDHVGEKAN** **CIVDSGTSAI** **TVPTDLNKML** **QNLDLCNNSK** **TFEF**

PF3D7_1407900 **IEYEGQLTYE** **KLNHDLYWQV** **DLDHFGEKAT** **AIVDSGTSSI** **TAPTELNKFF** **EGLDTCNNPK** **TLEF**

SN3_00700635 **YDVDGPIVYT** **PLVDKRYWMI** **NSQRVKSKTT** **VIIDSGTTLI** **AGPLDVDHIA** **HMFGSCDEAL** **DIAL**

NCLIV_022920 **VDFIGDINFS** **KVVDSRYWMI** **DTKKSNAPTT** **MIIDSGTSLI** **AGPLDVKRIA** **NMMGSCDKEK** **DLQL**

HHA_201840 **VDFIGDINFS** **PVVDSRYWMI** **NTKKSDAPTT** **MIIDSGTSLI** **VGPLDVKRIA** **TMMGSCEKAK** **DLQL**

TGME49_201840 **VDFIGDINFS** **PVVDSRYWMI** **NTKKSDAPTT** **MIIDSGTSLI** **VGPLDVKRIA** **TMMGSCEKAK** **DLQL**

Cvel_21827 **YDDPEDVQWI** **PLESEDYWTV** **KLGSIGGQGD** **CIVDSGTSLL** **AGPTAVGKIA** **KSIGQCSKKS** **DLKV**

Cvel_10378 **TNHDGPIRFT** **PLISESYWLV** **KMTLIDLTRP** **AIVDSGTSII** **LGPLPVTKIM** **ERMRPCSRKA** **DIHF**

BBBOND_0108020 **AAHKGHIRMI** **PVVTGYYWQV** **ELRWLGCDGA** **AIFDTGTAFN** **SMPYDFVQLM** **DYLSESESGQ** **IIRY**

cgd6_660 **IDFEEELHIL** **PVIREHYWQV** **ELEYIGCDYG** **VIFDSGTSFY** **TLPNFYNHFI** **KEYQDCSQIH** **NITY**

TA02750 **IDYKGDLYML** **PVIRELYWEI** **KLYWIGCNNE** **IIFDSGTSFN** **TMPHTFLLFK** **QYIKYCNGLE** **IIKY**

TOT_010000649 **VDCDSDVVML** **PVVREYYWEI** **KLKWVGCTEE** **VIFDSGTSFN** **TMPHDFMEFK** **KIVSSCHNPV** **IIKY**

PBANKA_122250 **ISYEGDIYML** **PVVKEYYWEV** **KLDYIGCEEE** **AIFDSGTSYN** **TMPSTISNFF** **KIVSPCNEEN** **TIKY**

PF3D7_0808200 **ISYEGDIYML** **PVLKESYWEV** **KLDYIGCDEE** **VIFDTGTSYN** **TMPSSMKTFL** **NLIHACTEQN** **IIKY**

PVX_088125 **VSYEGSIYML** **PVVKEYYWEV** **ELDYVGCEEK** **AIFDTGTSYN** **TMPSAMKGFF** **DVVPPCTEEN** **VIKY**

PVX_085030 **VEFEGDIYMF** **PVVREYYWEI** **QFDYIDCDSS** **LIFDSGTSFN** **SVPKSIGYFF** **KVVPKCDDSN** **NLTY**

PBANKA_101450 **VEFEGNIYMF** **PVVREYYWEI** **KFDYIDCDNN** **LIFDSGTSFN** **SVPKSIKYFF** **KVVPKCDANN** **NLTY**

PF3D7_1430200 **VDFEGDIYMF** **PVVKEYYWEI** **HFDYIDCGVN** **LIFDSGTSFN** **SVPKDIEYFF** **RVVPKCDDSN** **NLTY**

TOT_030000542 **ANYEGELKMF** **PVVREHYWEV** **ALDYLGCKEK** **LIFDSGTSLN** **SVPSSYSDFL** **SMIDSCFRSA** **SPTC**

BBBOND_0400170 **IDYLGEIQMF** **PVVREHYWEV** **KLDYVGCCDG** **LIFDSGTSLN** **TIPSSFGHFM** **SYFETCDVGA** **TITY**

GjASP3 **VDYEAPIHMF** **PVTQEHYWQM** **SLDYVGCDKK** **VILDSGTSFN** **TLPSNISSFL** **NLIPECPLDH** **SIKY**

SN3_00400750 **VDFEPPIHMF** **PVSREHYWET** **DLDYIGCDTP** **VILDSGTSFN** **TMPGKMRRLM** **EMIPECDLDD** **PITY**

ETH_00008525 **VDYESPIHMF** **PVEREHYWEV** **ALDHVGCEEG** **VILDSGTSFN** **TMPGNMRSFL** **EMIPECGDDD** **DITY**

NCLIV_063340 **VDYEPPIHMF** **PVTREHYWET** **PLDYIGCDEG** **VILDSGTSFN** **TMPSGLGKLL** **EMIPECDLED** **TITY**

HHA_246550 **VDYEAPIHMF** **PVTREHYWET** **SLDYIGCEEG** **VILDSGTSFN** **TMPSGLGKLL** **EMIPECNLDD** **TITY**

TGME49_246550 **VDYEAPIHMF** **PVTREHYWET** **SLDYIGCEEG** **VILDSGTSFN** **TMPSGLGKLL** **DMIPECNLDD** **TITY**

cgd1_2240 **QDKRNHVYWV** **PIIYPSVYKV** **SLESIGLLEE** **AIVDVGSTYS** **FFPSNYNKII** **NKFSLCFSDP** **IMNI**

TA17685 **YDKKSDMIWT** **PMVKSEFYIV** **RVFTIDVTDV** **FVLDTGTTLS** **TFEKEFIKIE** **KPIKICFSDI** **IITI**

TOT_030000196 **VDNTTQLIWA** **PLVKSEFYII** **KVLSFQEFKN** **FVLDTGTTIS** **TLEKEFNKIH** **KIFEKCTVDK** **SIVL**
