# Supplementary material for: A druggable secretory protein maturase of Toxoplasma essential for invasion and egress
Source: eLife. 2017 Sep 12;6:e27480. doi: 10.7554/eLife.27480 (PMC5595437; doi:10.7554/eLife.27480)
Supplement: Transparent reporting form [file elife-27480-transrepform.pdf]

1st Floor
24 Hills Road
Cambridge CB2 1JP, UK

P 01223 855340
W elifesciences.org
T @elife

eLife's transparent reporting form

We encourage authors to provide detailed information *within their submission* to facilitate the interpretation and replication of experiments. If you have any questions, please contact us: editorial@elifesciences.org.

Sample-size estimation

- You should state whether an appropriate sample size was computed when the study was being designed
- You should state the statistical method of sample size computation and any required assumptions
- If no explicit power analysis was used, you should describe how you decided what sample (replicate) size (number) to use

Please outline where this information can be found within the submission (e.g., page numbers or figure legends), or explain why this information doesn't apply to your submission:

Sample size estimation was not performed. All the experiments were performed in triplicate independent experiments in accordance with well-established reporting procedures for these assays. For the TAILS analysis, 4 sets of biological replicates (-ATc and +ATc 48h) were analyzed by mass spectrometry.

Replicates

- You should report how often each experiment was performed
- You should include a definition of biological versus technical replication
- The data obtained should be provided and sufficient information should be provided to indicate the number of independent biological and/or technical replicates
- If you encountered any outliers, you should describe how these were handled
- Criteria for exclusion/inclusion of data should be clearly stated
- High-throughput sequence data should be uploaded before submission, with a private link for reviewers provided (these are available from both GEO and ArrayExpress)

Please outline where this information can be found within the submission (e.g., page numbers or figure legends), or explain why this information doesn't apply to your submission:

eLife Sciences Publications, Ltd is a limited liability non-profit non-stock corporation incorporated in the State of Delaware, USA, with company number 5030732, and is registered in the UK with company number FC030576 and branch number BR015634 at the address 1st Floor, 24 Hills Road, Cambridge CB2 1JP | August 2014

The results for the Intracellular growth assay, Invasion Assays and Egress assays are shown as mean (+/- standard deviation) of three independent replicate experiments. The data value for each independent replicate experiment is taken as the average of three technical replicates. This has been reported in the Material and Methods section (under the description for each assay) as well as in the Figure legends of the corresponding assays.
Intracellular growth assays – Figure 1E and Figure 6-figure supplement 1A : Page 33 and corresponding figure legends
Invasion assays – Figure 1G and Figure 6C,I : Page 31 and corresponding figure legends
Egress assays – Figure 1H and Figure 6D,J : Page 31 and corresponding figure legends
MJ quantification – Figure 1L : Page 33 and corresponding figure legend
Host cell attachment assay – Figure 1F : Page 34 and corresponding figure legend
IC50 – Figure 6B : Page 38
Cleavage assays – Figure 7D,E,F and Figure 7-figure supplement 1B : Page 39

Statistical reporting

- Statistical analysis methods should be described and justified
- Raw data should be presented in figures whenever informative to do so (typically when N per group is less than 10)
- For each experiment, you should identify the statistical tests used, exact values of N, definitions of center, methods of multiple test correction, and dispersion and precision measures (e.g., mean, median, SD, SEM, confidence intervals; and, for the major substantive results, a measure of effect size (e.g., Pearson's r, Cohen's d)
- Report exact p-values wherever possible alongside the summary statistics and 95% confidence intervals. These should be reported for all key questions and not only when the p-value is less than 0.05.

Please outline where this information can be found within the submission (e.g., page numbers or figure legends), or explain why this information doesn't apply to your submission:

Interpretation of the TAILS dataset is described in the Materials and Methods sections on Page 34-38

(For large datasets, or papers with a very large number of statistical tests, you may upload a single table file with tests, Ns, etc., with reference to page numbers in the manuscript.)

Additional data files ("source data")

- We encourage you to upload relevant additional data files, such as numerical data that are represented as a graph in a figure, or as a summary table
- Where provided, these should be in the most useful format, and they can be uploaded as "Source data" files linked to a main figure or table
- Include model definition files including the full list of parameters used
- Include code used for data analysis (e.g., R, MatLab)
- Avoid stating that data files are "available upon request"

Please indicate the figures or tables for which source data files have been provided:

Source data files in GraphPad format have been provided as

Figure 1 – Source data 1.xlsx: Figure 1 (Panels E,F,G,H,L)
Figure 6 – Source data 1.xlsx : Figure 6 (Panels C,D,I,J) and Figure 6-figure supplement 1 (Panel A)
Figure 7 – Source data 1.xlsx : Figure 7 (Panels D,E,F) and Figure 7-figure supplement 1 (Panel B)

The following GraphPad files have also been deposited under the compressed zip file Graphs_Source_Data.zip
Figure 1 ASP3_Graphs.pzf : Figure 1 (Panels E,F,G,H,L)
Figure 6 IC50.pzf : Figure 6 (Panel B)
Figure 6 49c Assays.pzf : Figure 6 (Panels C,D,I,J) and Figure 6-figure supplement 1 (Panel A)
Figure 7 Cleavage Assays.pzf : Figure 7 (Panels D,E,F) and Figure 7-figure supplement 1 (Panel B)

Figure 2 and Table 1, Table supplements 1-5:
The source data from the LC-MS-MS datasets generated by TAILS experiment has been deposited on Proteome Xchange Consortium via the PRIDE (http://proteomecentral.proteomexchange.org/cgi/GetDataset) partner repository with the dataset identifier **PXD006235**.
